# Gastric, pancreatic, and ureteric duplication

**DOI:** 10.4103/0971-9261.69138

**Published:** 2010

**Authors:** Anindya Chattopadhyay, S. K. Mitra, Soumitra Dutta, Hema Chakraborty

**Affiliations:** Susruta Clinic and Research Institute for Advanced Medicine, JC-16 & 17, Salt Lake City, Kolkata – 700 098, India

**Keywords:** Gastrointestinal duplications, pancreatic duplications, ureteral atresia, ureteric duplication

## Abstract

We report a case of an 8-month-old, asymptomatic child who was incidentally detected to have two cystic structures in the abdomen. Surgical exploration revealed a gastric and pancreatic duplication cyst along with a blind-ending duplication of the right ureter. Excision of the duplications was relatively straightforward, and the child made an uneventful recovery. This constellation of duplications has not been reported before.

## INTRODUCTION

Coexistence of gastric duplications and pancreatic anomalies have rarely been reported, and they can lead to a variety of symptoms, such as recurrent abdominal pain, vomiting, or pancreatitis.[1–3] We recently had an occasion to operate on an asymptomatic child with a gastric and pancreatic duplication cyst, who in addition also had a blind-ending duplication of the right ureter.

## CASE REPORT

**Figure 1:**
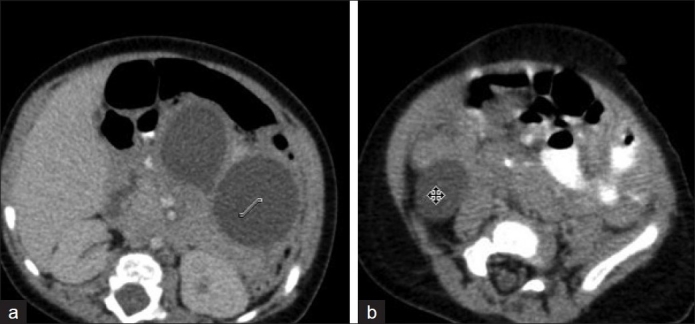
CT scan. (a) Dumbbell-shaped retrogastric cyst. (b) Separate cyst in the right iliac fossa

An 8-month-old, asymptomatic male child presented with a history of incidentally detected abdominal cysts on ultrasound scan done elsewhere a month and a half earlier. Contrast-enhanced computed tomogram (CECT) scan of the abdomen also confirmed the presence of a dumbbell-shaped cystic mass located posterior to the stomach with a separate cystic mass located in the right iliac fossa (RIF) [Figure 1].

On examination, the child was cheerful, active, and weighed 9 kg. Physical examination revealed a nontender 7 × 5cm mass in the left upper quadrant, which had restricted mobility, and a 5 × 2 cm nontender mass in the RIF. On investigation, his Hb was found to be 12 gm%, had normal total and differential leukocyte counts, electrolytes, and renal function tests. We reviewed the CECT scans and concluded that the cyst in the left upper abdomen could be a gastric duplication or a lymphatic cyst, while the latter etiology seemed most likely for the cyst in the RIF. Both the kidneys appeared normal on CECT.

At surgery, there was a dumbbell-shaped cyst arising from the greater curvature of the stomach, burrowing into the transverse mesocolon and also into the stomach bed. The cyst was dissected off from the transverse mesocolon and a tongue of pancreatic tissue draped over the isthmus of the dumbbell was found, at a right angle to the normal position of the pancreas. This tongue was dissected off, and fell back into a normal anatomic position of the pancreas. The cyst was then enucleated from the musculature of the greater curvature without breaching the gastric lumen. Through the same incision, the RIF was explored and a tubular cystic structure was found with a blind end close to the internal iliac vessels. Superiorly the cyst was arising from the surface of the lower pole of the right kidney, but without any apparent pelvic connection. Further dissection revealed a normal right-sided pelvis and normal ureter going toward the bladder from which the cyst was entirely separate. The cyst was transfixed at the level of the kidney and excised.

The postoperative course was unremarkable and the child was discharged on the 5^th^ postoperative day. A histologic examination revealed that the upper abdominal dumbbell-shaped cyst had two components: the anterior half of the cyst had the characteristics of a gastric duplication, whereas the posterior cyst was lined by sparse epithelium containing pancreatic tissue and the wall had fibrous tissue. The cyst attached to the kidney was lined with urothelium, had a wall of fibrous tissue, and was a ureteric duplication.

## DISCUSSION

Gastrointestinal duplications are rare and interesting anomalies because of their association with other malformations and possibly varied embryopathogenesis. Gastric and duodenal duplications have been rarely reported to be associated with accessory pancreas and pancreatic duplications.[3–5] In one such report, the patient also had a duplication of the left ureter.[5]

Arising as a diverticulum from the foregut, pancreatic developmental anomalies include bifid pancreas, annular pancreas, accessory pancreas, and anomalies of the pancreatic ductal system. Pancreatic duplications present as cysts in relation to the organ with a wall containing muscle lined by pancreatic or gastric epithelium.[1] Variable inflammatory response can destroy the lining and lead to confusion with idiopathic pseudocysts.[1] These duplications are believed to occur either as a result of nonregressing diverticula from the pancreatic bud[1,6] or as a result of traction by a neuroenteric band.[5] The latter etiology may explain the coexistence of gastric and pancreatic duplications.

Although usually asymptomatic in younger infants, gastric–pancreatic duplications cause symptoms from their mass effect leading to gastric outlet obstruction or from pain and ulceration due to inflammation within the cyst.[1,7] Erosion into neighboring viscera can lead to gastrointestinal hemorrhage. Gastric and duodenal duplications that communicate with the pancreatic ductal system may give rise to abdominal pain and/or pancreatitis.[1–3] Imaging studies such as ultrasound scan and CECT have replaced the earlier investigations of upper gastrointestinal radiographs and barium enemas, and allow a presumptive diagnosis to be made as in this case from the location of the cyst.

Surgical extirpation of the cyst is often possible without entering the gastric lumen.[5] Adherent pancreas should primarily be dissected off if possible to visualize clearly the coexisting pancreatic anatomy and abnormality before considering any pancreatic resection.[1,5] The need for major resectional surgery is rare.

Blind-ending ureteral duplications are the rarest of upper urinary tract anomalies[8] and are differentiated from ureteral diverticulum by the criteria developed by Culp.[9] Blind-ending inverted Y duplications may arise as a result of abnormal canalization of the ureteral bud, absorption of the common stem of the mesonephric duct into the bladder, or fusion of two ureteral buds before joining the metanephric blastema.[10] Such duplications may be connected to the urinary tract only by fibrous cords,[10] or their communication with the urinary tract may be totally lost.[8] In these cases, the diagnosis of the ureteric duplication is not possible by intravenous pyelography or retrograde studies and a nonenhancing mass is seen on CECT scan.[10] The differential diagnoses in these cases include mesenteric cysts, omental cysts, or gastrointestinal duplications. These duplications may come to attention by the presence of an abdominal mass, abdominal pain, recurrent urinary tract infections, hematuria, or lithiasis.[11] Surgical excision of the symptomatic lesion is recommended[10] and as in our case may provide the only means to diagnose the lesion. Depending on the relationship of the duplication to the normal ureter, excision can be simple or complicated, always keeping the vascularity of the normal ureter safe.
